# Optimization and impact of sensitivity mode on abbreviated scan protocols with population-based input function for parametric imaging of [^18^F]-FDG for a long axial FOV PET scanner

**DOI:** 10.1007/s00259-024-06745-3

**Published:** 2024-05-20

**Authors:** W. Lan, H. Sari, A. Rominger, C. la Fougère, F. P. Schmidt

**Affiliations:** 1 Department of Nuclear Medicine and Clinical Molecular Imaging, University Hospital Tuebingen, Tuebingen, Germany; 2 Advanced Clinical Imaging Technology, Siemens Healthineers International AG, Lausanne, Switzerland; 3 Department of Nuclear Medicine, Inselspital, Bern University Hospital, University of Bern, Bern, Switzerland; 4 Cluster of Excellence iFIT (EXC 2180) “Image Guided and Functionally Instructed Tumor Therapies”, University of Tuebingen, Tuebingen, Germany; 5 Werner Siemens Imaging Center, Department of Preclinical Imaging and Radiopharmacy, University of Tuebingen, Tuebingen, Germany

**Keywords:** LAFOV PET, Total-body PET, Parametric imaging, Patlak, Population-based input function

## Abstract

**Background:**

The long axial field of view, combined with the high sensitivity of the Biograph Vision Quadra PET/CT scanner enables the precise deviation of an image derived input function (IDIF) required for parametric imaging. Traditionally, this requires an hour-long dynamic PET scan for [^18^F]-FDG, which can be significantly reduced by using a population-based input function (PBIF). In this study, we expand these examinations and include the scanner’s ultra-high sensitivity (UHS) mode in comparison to the high sensitivity (HS) mode and evaluate the potential for further shortening of the scan time.

**Methods:**

Patlak K_i_ and DV estimates were determined by the indirect and direct Patlak methods using dynamic [^18^F]-FDG data of 6 oncological patients with 26 lesions (0–65 min p.i.). Both sensitivity modes for different number/duration of PET data frames were compared, together with the potential of using abbreviated scan durations of 20, 15 and 10 min by using a PBIF. The differences in parametric images and tumour-to-background ratio (TBR) due to the shorter scans using the PBIF method and between the sensitivity modes were assessed.

**Results:**

A difference of 3.4 ± 7.0% (K_i_) and 1.2 ± 2.6% (DV) was found between both sensitivity modes using indirect Patlak and the full IDIF (0–65 min). For the abbreviated protocols and indirect Patlak, the UHS mode resulted in a lower bias and higher precision, e.g., 45–65 min p.i. 3.8 ± 4.4% (UHS) and 6.4 ± 8.9% (HS), allowing shorter scan protocols, e.g. 50–65 min p.i. 4.4 ± 11.2% (UHS) instead of 7.3 ± 20.0% (HS). The variation of K_i_ and DV estimates for both Patlak methods was comparable, e.g., UHS mode 3.8 ± 4.4% and 2.7 ± 3.4% (K_i_) and 14.4 ± 2.7% and 18.1 ± 7.5% (DV) for indirect and direct Patlak, respectively. Only a minor impact of the number of Patlak frames was observed for both sensitivity modes and Patlak methods. The TBR obtained with direct Patlak and PBIF was not affected by the sensitivity mode, was higher than that derived from the SUV image (6.2 ± 3.1) and degraded from 20.2 ± 12.0 (20 min) to 10.6 ± 5.4 (15 min). K_i_ and DV estimate images showed good agreement (UHS mode, RC: 6.9 ± 2.3% (K_i_), 0.1 ± 3.1% (DV), peak signal-to-noise ratio (PSNR): 64.5 ± 3.3 dB (K_i_), 61.2 ± 10.6 dB (DV)) even for abbreviated scan protocols of 50–65 min p.i.

**Conclusions:**

Both sensitivity modes provide comparable results for the full 65 min dynamic scans and abbreviated scans using the direct Patlak reconstruction method, with good K_i_ and DV estimates for 15 min short scans. For the indirect Patlak approach the UHS mode improved the K_i_ estimates for the abbreviated scans.

**Supplementary Information:**

The online version contains supplementary material available at 10.1007/s00259-024-06745-3.

## Introduction

Hybrid positron emission tomography (PET)/computed tomography (CT) systems have been accepted as standard-of-care imaging modality in oncology, cardiology and neurology [1, 2]. The standardised uptake value (SUV), determined from a static image at 60 min p.i., is commonly used with [^18^F]-FDG and enables semi-quantitative image analysis [3, 4]. However, kinetic modelling to better distinguish between specific and unspecific tracer uptake potentially provides a more accurate assessment of the true metabolic activity of the tumour and can therefore be advantageous for diagnosis, therapeutic response monitoring and drug development [5–8].

Pharmacokinetic models require an accurate knowledge of the arterial input function (AIF), i.e., the time-dependent concentration of the radiotracer in the arterial blood. Traditionally, this requires long scan times of up to 60 min due to the kinetics of [^18^F]-FDG [9], as well as additional invasive arterial blood sampling during the entire PET scan. A non-invasive method, which therefore can be easier applied in clinical routine, is to obtain an image-derived input function (IDIF). Large blood pools are preferred to obtain the IDIF over small structures, such as the carotid arteries often used in brain PET studies [10], as the partial volume effect degrades the quantification.

Long axial field of view (LAFOV) PET/CT scanners can image 106–198 cm [11–13] of the body in a single bed position and thus derive the IDIF from large vascular structures or blood vessels [14, 15]. In addition, the increased sensitivity of up to 176 kcps/MBq [11] of these scanners enables an accurate quantification even for short frame durations, resulting in a high temporal resolution for dynamic PET scans [11–13]. Furthermore, the superior time resolution down to 228 ps [11] provides precise time-of-flight (TOF) information, which translates into an identification of the annihilation position with an accuracy of 3.4 cm full-width-at-half-maximum (FWHM). Incorporating this localization as TOF weighting process into the image reconstruction reduces the propagation of statistical noise in the image [16]. This TOF gain can be considered as a virtual sensitivity amplifier that further increases the effective sensitivity of the scanner (e.g. according to Conti et al. [17] a TOF gain of 6 for an object size of 30 cm and 228 ps time resolution).

Despite these advances in sensitivity, the hour-long scan time required to obtain the full-length IDIF still causes patient discomfort and occupies critical infrastructure, limiting its use in routine clinical practice. Abbreviated scans provide only a partial IDIF, but can be used for parametric imaging when combined with a population-based input function (PBIF) [18, 19]. Using this method together with the excellent spatial-temporal resolution of LAFOV PET/CT scanners, it has been shown that kinetic microparameters, such as tracer net influx rate (K_i_) and tracer distribution volume (DV) can be obtained with a high accuracy for 20 min short scans [20–22]. However, the studies by Sari et al. [20] and Sluis et al. [21] using the Biograph Vision Quadra LAFOV PET/CT (Siemens Healthineers, Knoxville, TN, USA) scanner used only a fraction of the acquired event data as the clinical reconstruction software at that time limited the acceptance angle to 18° (high sensitivity (HS) mode). In terms of sensitivity, the full acceptance angle of 52° (ultra-high sensitivity (UHS) mode) is desired as it increases the sensitivity from 83 kcps/MBq to 176 kcps/MBq [11].

Several studies have recently been performed to assess the impact of the UHS mode on quantification, spatial resolution, partial volume effect and image quality for different isotopes [14, 23, 24]. Another study elaborated on the potential for dose reduction in static [^18^F]-FDG scans using the maximum acceptance angle [25]. However, to date, these examinations have only been performed for static PET scans. Therefore, in this study we evaluate the impact on parametric imaging using the UHS mode of the Biograph Vision Quadra with 65 min long dynamic scans. We evaluated Patlak K_i_ and DV estimates of lesions from 6 oncological patients for two different approaches to derive the tracer kinetics. Either obtained by region-of-interest linear Patlak modelling [26] or by incorporating the Patlak model directly into the image reconstruction process [27] to obtain parametric images. The first aim of this study was to determine the impact of the sensitivity mode on parametric imaging when using the full patient-individual IDIF of 65 min. Further we extended these examinations to abbreviated scan durations of 20 min and less, using partial IDIFs combined with a PBIF. For these studies we aim to determine the optimum settings for parametric image reconstruction in terms of the number of Patlak frames.

Short scan times of 20 min or less may facilitate the integration of parametric imaging into clinical routine. Therefore, the final aim is to elaborate whether a further reduction in scan time to less than 20 min could be achieved by using the UHS mode, due to its ability to maintain high event statistics at short frame durations.

## Materials and methods

### Subjects and imaging protocol

This work includes [^18^F]-FDG PET data from a clinically heterogenous group of 6 oncological subjects (4 females and 2 males; mean age: 60 ± 20 years, mean weight: 76.1 ± 28.2 kg). The subjects were scanned as part of a dynamic imaging protocol, where PET emission data were acquired for 65 min with a Biograph Vision Quadra LAFOV PET/CT scanner. The administration of [^**18**^F]-FDG (mean activity: 240.6 ± 82.8 MBq) was performed approximately 15 s after the start of PET acquisition. PET data were recorded for all possible line of response (LORs) for the maximum acceptance angle of 52°. Following the PET scan, a low-dose CT scan was used for anatomical information and PET data corrections.

### Image reconstruction, image-derived input function and lesion delineation

Image reconstruction was performed using an investigational software prototype (e7 tools, Siemens Healthineers). A standard clinical reconstruction protocol was employed using an Ordinary-Poisson Ordered-Subsets Expectation-Maximization (OP-OSEM) algorithm with four iterations and five subsets, point-spread-function (PSF) modelling and using TOF information. Images were reconstructed into a matrix of 440 × 440 × 645 with 1.65 × 1.65 × 1.65 mm^3^ voxel size and no filter was applied.

Image reconstruction was performed with either all LORs (acceptance angle 52°) in the ultra-high sensitivity (UHS) or in the high sensitivity (HS) mode using a subset of the LORs (acceptance angle 18°).

Another investigational software prototype, (SnakeVOI, Siemens Healthineers) was used to automatically obtain the volume of interest (VOI) required to obtain the patient-specific image-derived input function (IDIF). A high accuracy for the IDIF obtained by SnakeVOI, in terms of a low deviation of the area under the curve (AUC) of 3 ± 6% in comparison to arterial blood sampling has been reported [5]. In a first step this software performs an automatic aorta landmarking in the CT image using a learning-based algorithm for automatic medical image annotation [28]. In a second step a “snake-shaped” VOI (see Fig. 7c) is automatically generated in the descending thoracic aorta. This VOI (volume: 2.13 ± 0.51 cm^3^) derived from the CT image was transferred to the dynamic PET frames serving as a mask to determine the mean activity concentration in the descending aorta over time to obtain the IDIF. By using SnakeVOI, the automatic aorta landmarking was successfully performed for all 6 patients (see Supplemental Fig. 1) without the need of manual corrections.

The delineation of 26 lesions was performed by an experienced nuclear medicine physician using an iso-contour tool (PMOD 4.1, PMOD Technologies, Zurich, Switzerland), with a threshold set to 50% of the maximum values.

To assess the impact of the sensitivity mode on the IDIF, IDIFs were compared for all patients reconstructed in UHS and HS mode. The PET data was framed to 2 × 10 s, 30 × 2 s, 4 × 10 s, 8 × 30 s, 4 × 60 s, 5 × 120 s and 9 × 300 s and the area under the curve (AUC) values for the IDIFs were obtained. As quantitative metric for comparison the mean bias, defined as relative difference between AUC obtained by HS and UHS mode, and the precision, defined as the standard deviation of the bias, were determined.

### Indirect Patlak

To evaluate the impact of the sensitivity mode on parametric imaging, Patlak slope K_i_ (influx rate) and intercept DV (distribution volume) estimations were determined by the linear Patlak model [21, 29].

1 $$\frac{c\left({t}_{n}\right)}{{C}_{p}\left({t}_{n}\right)}= {K}_{i}\frac{{\int }_{0}^{t}{c}_{p}\left(\tau \right)d\tau }{{C}_{p}\left({t}_{n}\right)}+DV,{ t}_{n}>{t}^{*}, n=1\dots N$$

where $c\left(t\right)$ is the measured activity concentration of the time activity curve at each voxel, ${c}_{p}\left(t\right)$ is the parameterized blood input function, and${t}^{*}$ is the time when the kinetic model reaches the stable state.${ t}_{n}$ with $, n=1\dots N$ represents the mid-time points for the $N$ dynamic PET frames.

The Patlak fit for K_i_ and DV estimation was performed for t*= 45, 50, 55 min [20] for scan durations of 45–65 min, 50–65 min and 55–65 min, respectively, using MATLAB v2023b (MathWorks Inc., Natick, MA, USA).

Of note, in order to study abbreviated scan durations of 20 min or less we evaluated only t* ≥20 min, whereas for t*=20 min a high precision of 13% in Ki estimate in comparison to t*=35 min was reported by Sari et al. [20].

Throughout this work, this approach of VOI-based kinetic modelling is referred to as *indirect Patlak*. K_i_ and DV values for the segmented lesions were determined for both sensitivity modes and a linear regression was used to assess the correlation between both modes.

Further, bias and precision for K_i_ and DV as relative difference between UHS and HS mode were obtained and reported according to the respective lesions size and position. The position of the lesion was determined as distance of the lesion VOI centroid to the centre of the axial field of view (aFOV).

### Impact of Patlak frames on IDIF based indirect Patlak for both sensitivity modes

The impact of the frame duration (or number of frames) used to determine Patlak K_i_ and DV estimates was evaluated. The first 45 min of the PET data were framed to 2 × 10 s, 30 × 2 s, 4 × 10 s, 8 × 30 s, 4 × 60 s, 5 × 120 s and 5 × 300 s. For the 45 min (t*) to 65 min data a different number of frames – denoted as *Patlak frames* in this work – were used for image reconstruction, IDIF determination and indirect Patlak analysis: 4 × 300 s, 5 × 240 s, 6 × 200 s, 8 × 150 s, 10 × 120 s, 12 × 100 s, 15 × 80 s, 20 × 60 s, 25 × 48 s, 30 × 40 s, 40 × 30 s. K_i_ and DV were determined for both sensitivity modes and bias and precision were reported with reference values obtained for 4 Patlak frames and the respective sensitivity mode.

### Impact of sensitivity mode on sPBIF based indirect Patlak for different t*

Next the impact of the sensitivity mode for indirect Patlak estimates was evaluated when using partial IDIFs, such as obtained from abbreviated scan protocols from 45-65 min (t*=45 min), 50–65 min (t*=50 min) and 55–65 min p.i. (t*=55 min). The AUC of the last 10 min (55–65 min p.i.) tail of the patient-individual partial IDIF were used to scale a population-based input function (PBIF), as determined by Sari et al. [20], to obtain patient-individual scaled PBIFs (sPBIFs). PET data were framed to 2 × 10 s, 30 × 2 s, 4 × 10 s, 8 × 30 s, 4 × 60 s, 5 × 120 s and 9 × 300 s, resulting in either 2 (t*=55 min), 3 (t*=50 min) or 4 (t*=45 min) Patlak frames of 300 s. As a reference to determine the bias and precision, the K_i_ and DV estimates obtained with the full IDIF (t*=45 min and 8 Patlak frames) in the respective sensitivity mode were used. Of note, 8 Patlak frames were used as a reference here instead of 4 as in the previous section, as 8 frames showed to be more robust in terms of K_i_ stability based on the results of the previous section.

### Impact of Patlak frames on sPBIF based indirect Patlak for abbreviated scan durations

For the sake of simplicity only the UHS mode was used to evaluate the impact of the number of Patlak frames on sPBIF based indirect Patlak estimates for abbreviated scan durations. PET data was reframed for the last 20, 15 and 10 min of the scans according to Table 1.

**Table 1:** Number of frames and duration for abbreviated scan protocols processed with indirect Patlak

PET data	t* [min]	Number of frames × Frame duration [s]								
45–65 min p.i.	45	–	–	4 × 300	5 × 240	6 × 200	8 × 150	10 × 120	12 × 100	15 × 80
50–65 min p.i.	50	–	3 × 300	4 × 225	5 × 180	6 × 150	8 × 112.5	10 × 90	12 × 75	15 × 60
55–65 min p.i.	55	2 × 300	3 × 200	4 × 150	5 × 120	6 × 100	8 × 75	10 × 60	12 × 50	15 × 40

### Direct Patlak

Parametric images K_i_ and DV were reconstructed using the direct Patlak method implemented in an investigational software prototype the e7 tools (Siemens Healthineers). The Direct Patlak reconstruction employs a nested expectation maximization algorithm [30] and images were reconstructed with 8 iteration and 5 subsets, PSF-TOF and a 2 mm FWHM Gaussian filter [31]. The previously obtained lesion VOIs were used to determine K_i_ and DV values directly from the parametric images. In the first step, the patient individual IDIF was used.

### Impact of Patlak frames on IDIF based direct Patlak for both sensitivity modes

In the same way as for the indirect Patlak also for the direct Patlak method the 45 min (t*) to 65 min data was reframed with a different number of frames: 4 × 300 s, 5 × 240 s, 6 × 200 s (the highest number of Patlak frames supported by the software was 6). Bias and precision for K_i_ and DV estimates were determined with the reference reconstruction using the respective sensitivity mode, t*=45 min and 4 × 300 s Patlak frames.

### Impact of Patlak frames on sPBIF based direct Patlak for abbreviated scan durations and both sensitivity modes

The sPBIF was obtained analogue as for the indirect Patlak method and for a scan duration from 45 to 65 min (t*=45 min) and K_i_ and DV estimates by direct Patlak reconstruction were assessed for 4,5 and 6 Patlak frames. The reference was set to the respective sensitivity mode and number of Patlak frames but patient-individual IDIF based direct Patlak reconstruction.

To evaluate the impact of the number of Patlak frames on sPBIF based direct Patlak estimates for abbreviated scan durations the last 20, 15 and 10 min of the scans according to Table 2 were used.

**Table 2:** Number of frames and duration for abbreviated scan protocols processed with direct Patlak

PET data	t* [min]	Number of frames × Frame duration [s]		
45–65 min p.i.	45	4 × 300	5 × 240	6 × 200
50–65 min p.i.	50	4 × 225	5 × 180	6 × 150
55–65 min p.i.	55	4 × 150	5 × 120	6 × 100

The reference was set to the respective sensitivity mode and number of Patlak frames but patient-individual IDIF based direct Patlak reconstruction.

### Patlak K_i_, DV image comparison for sPBIF based direct Patlak for abbreviated scan durations and both sensitivity modes

Parametric Patlak K_i_ and DV images obtained by the direct Patlak method were compared for the abbreviated scan durations using the sPBIF with the images using the patient-individual IDIF (t*=45 min, 45–65 min p.i.) as reference. In addition, the SUV image (60–65 min p.i.) obtained by the iterative reconstruction was shown for comparison. Quantitative evaluation was performed by determining non-absolute and absolute relative change (% RC), the structural similarity index measure (SSIM), peak signal-to-noise ratio (PSNR) and tumour-to-background ratio (TBR) was determined as ratio of the peak value for each lesion to the mean of a spherical VOI with 3 cm diameter placed in the liver.

### Statistical analysis

Linear regression analysis was used to assess the difference between lesion K_i_ and DV estimates obtained by indirect Patlak and patient individual IDIF for both sensitivity modes. Differences between K_i_ as well as DV bias for the indirect Patlak approach for each Patlak start time were assessed using the paired Student’s t-test. A two-way repeated measures ANOVA was performed to analyze the effect of sensitivity mode and number of Patlak frames on K_i_ and DV bias. Statistical significance was considered for p values less than 0.05. Statistical analysis and production of graphs were performed using SPSS Statistics, version 29.0 (IBM Crop., Armonk, NY, USA) and MATLAB v2023b (MathWorks Inc., Natick, MA, USA).

## Results

### IDIF comparison sensitivity modes

**Fig. 1:**
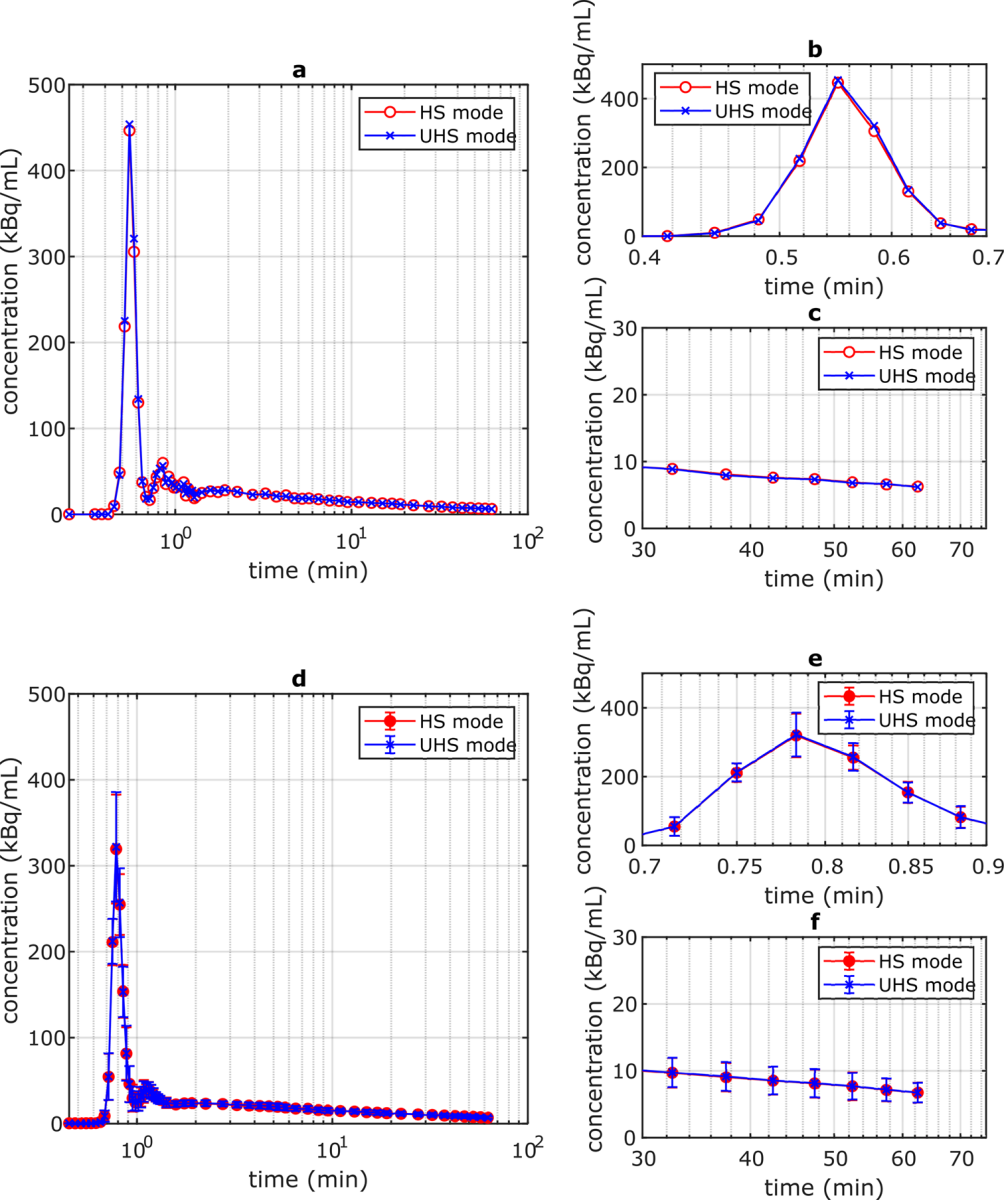
(**a**) Example of single patient IDIF obtained by HS and UHS mode; (**b**): enlarged view for the IDIF peak region; (**c**): enlarged view for 30–65 min p.i.; (**d**): Mean IDIF of 6 patients obtained by HS and UHS mode; (**e**) enlarged view for the mean IDIF peak region; (**f**) enlarged view of the mean IDIF for 30–65 min p.i

The IDIF obtained with HS and UHS mode showed a negligible difference (Fig. 1) which is in accordance with a low bias of the AUC values of -0.7 ± 0.4%.

### IDIF based indirect Patlak K_i_, DV for both sensitivity modes

**Fig. 2:**
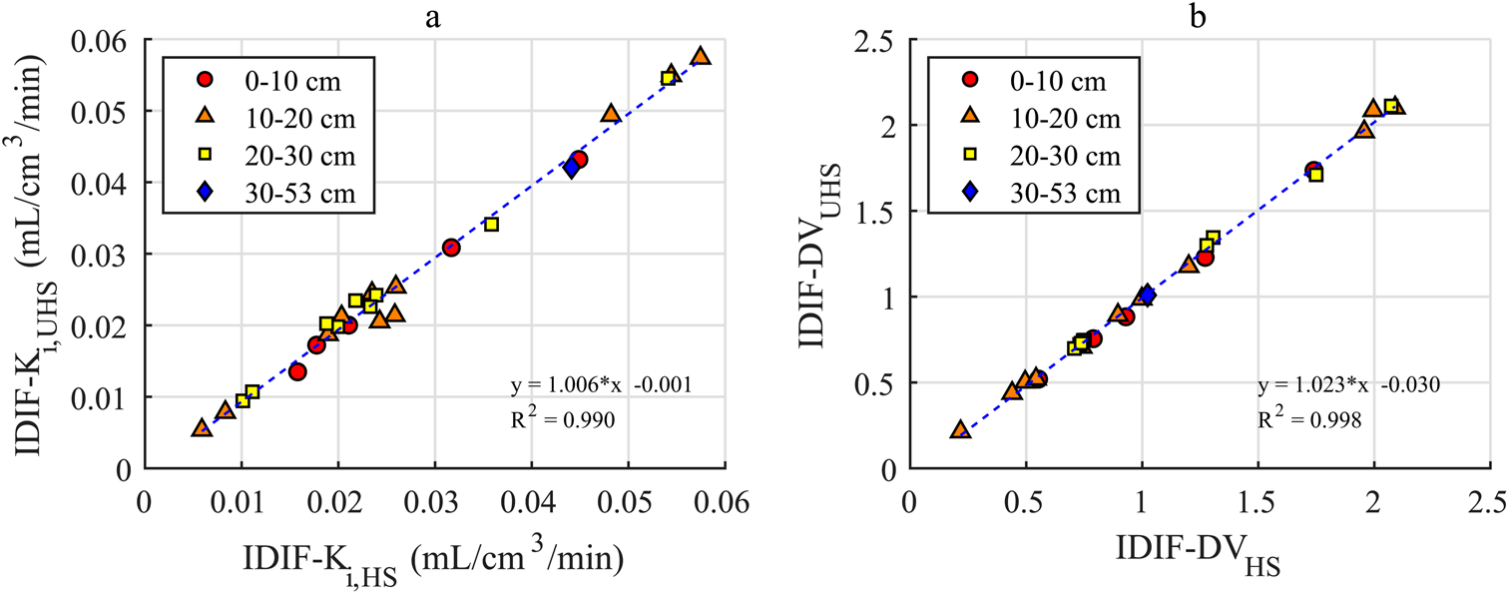
K_i_ (**a**) and DV (**b**) values obtained by HS and UHS mode with linear regression and axial position of lesions binned into intervals of 0–10 cm, 10–20 cm, 20–30 cm and 30–53 cm

The comparison of Patlak K_i_ and DV values in Fig. 2 shows a good agreement between HS an UHS mode with R^2^ = 0.990 and R^2^ = 0.998, respectively.

This is in accordance with the low bias and high precision of 3.4 ± 7.0% and 1.2 ± 2.6% for K_i_ and DV, respectively. No correlation between differences in K_i_ or DV and the size or axial position of the lesion could be observed (Supplemental Fig. 2).

### Impact of Patlak frames on IDIF based indirect Patlak for both sensitivity modes

**Fig. 3:**
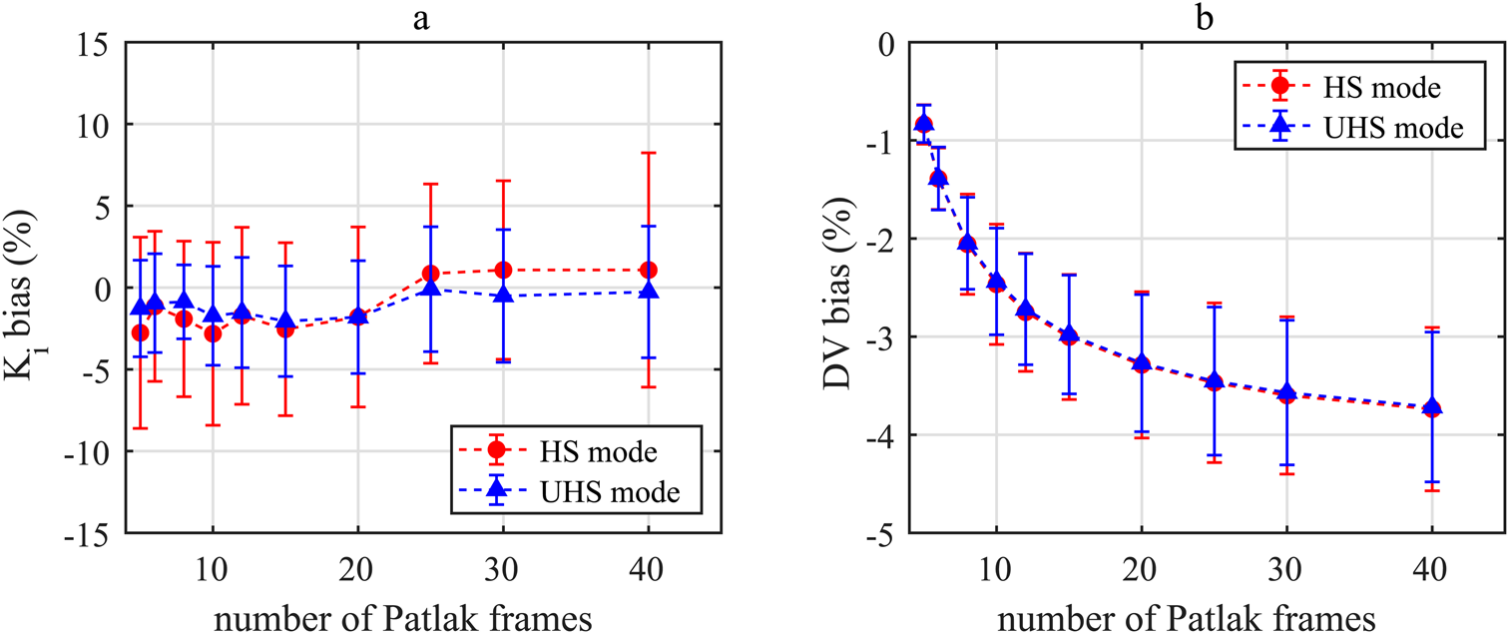
Bias and precision for K_i_ (**a**) and DV (**b**) values for both sensitivity modes and for different number of Patlak frames with reference to values obtained with 4 Patlak frames and t*=45 min

The bias for K_i_ remained stable between 5 and 20 Patlak frames (Fig. 3a) with a value of -2.1 ± 5.3% and -1.5 ± 3.1% for the HS and UHS mode, respectively. A higher precision was observed for the UHS mode for all Patlak frames, e.g., for 8 Patlak frames 2.3% (UHS) and 4.8% (HS). In contrast, the bias of DV decreased and the precision was lower for a higher number of Patlak frames (Fig. 3b).

Comparing K_i_ (Fig. 3a) and DV (Fig. 3b) bias with respect to the different number of Patlak frames, DV bias is less effected by the sensitivity mode for DV than for K_i_, e.g. for 8 Patlak frames DV bias is -2.0 ± 0.5% (HS) and − 2.0 ± 0.4% (UHS) whereas the difference for K_i_ estimates is higher 1.9 ± 4.8% (HS) and 0.9 ± 2.3% (UHS). For 30 Patlak frames, DV bias is -3.6 ± 0.8% (HS) and − 3.6 ± 0.7% (UHS) whereas the bias of K_i_ estimates is -1.1 ± 5.5% (HS) and − 0.5 ± 4.0% (UHS).

### Impact of sensitivity mode on sPBIF based indirect patlak for different t*

**Fig. 4:**
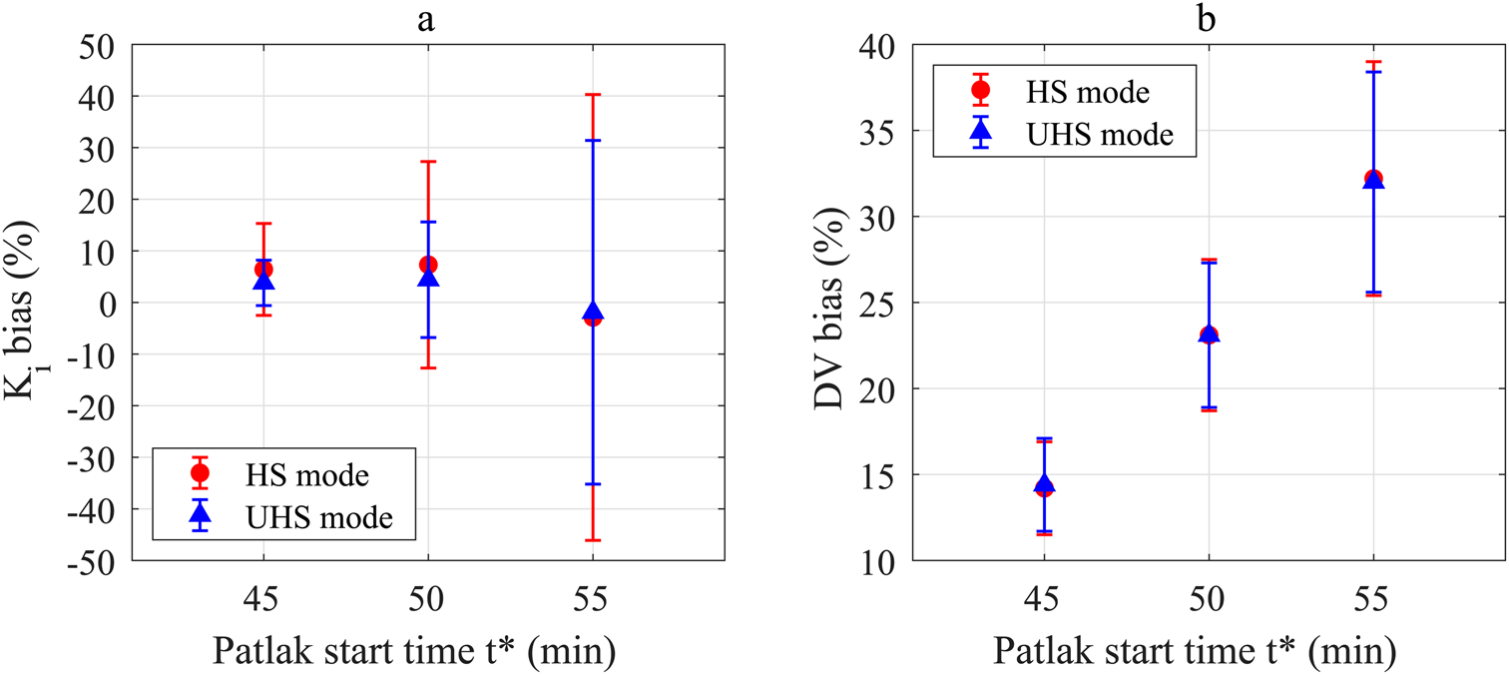
Bias and precision for K_i_ (**a**) and DV (**b**) values for indirect Patlak with sPBIF, both sensitivity modes, t*=45, 50 and 55 min with reference to IDIF based estimation (t*=45 min, 8 Patlak frames)

Figure 4 illustrates that the lowest bias and highest precision of K_i_ and DV could be obtained with the sPBIF with t*=45 min

A better precision for K_i_ estimates was determined for a lower t* for both sensitivity modes: HS: 8.9% (t*=45 min), 20.0% (t*=50 min) and 43.2% (t*=45 min); UHS: 4.4% (t*=45 min), 11.2% (t*=50 min) and 33.3% (t*=55 min). Similar results were obtained for DV estimates: HS: 2.7% (t*=45 min), 4.4% (t*=50 min) and 6.8% (t*=45 min); UHS: 2.7% (t*=45 min), 4.2% (t*=50 min) and 6.4% (t*=55 min).

Bias of K_i_ showed no significant difference between both sensitivity modes: t*=45 min: 6.4%/3.8% (HS/UHS, *p* = 0.10), t*=50 min: 7.3%/4.4% (HS/UHS, *p* = 0.28) and t*=55 min: -2.9%/-1.9% (HS/UHS, *p* = 0.88). Similar no significant difference was obtained between both sensitivity modes for DV estimates: t*=45 min: 14.2%/14.4% (HS/UHS, *p* = 0.16), t*=50 min: 23.1%/23.1% (HS/UHS, *p* = 0.99) and t*=55 min: 32.2%/32.0% (HS/UHS, *p* = 0.21). Of note, the lowest bias for DV estimates was obtained for t*=45 min, as this resembles the same t* as the reference IDIF based estimation with the same number of Patlak frames for linear regression (see Supplemental Fig. 3 for exemplary Patlak plot).

### Impact of Patlak frames on sPBIF based indirect Patlak for abbreviated scan durations

**Fig. 5:**
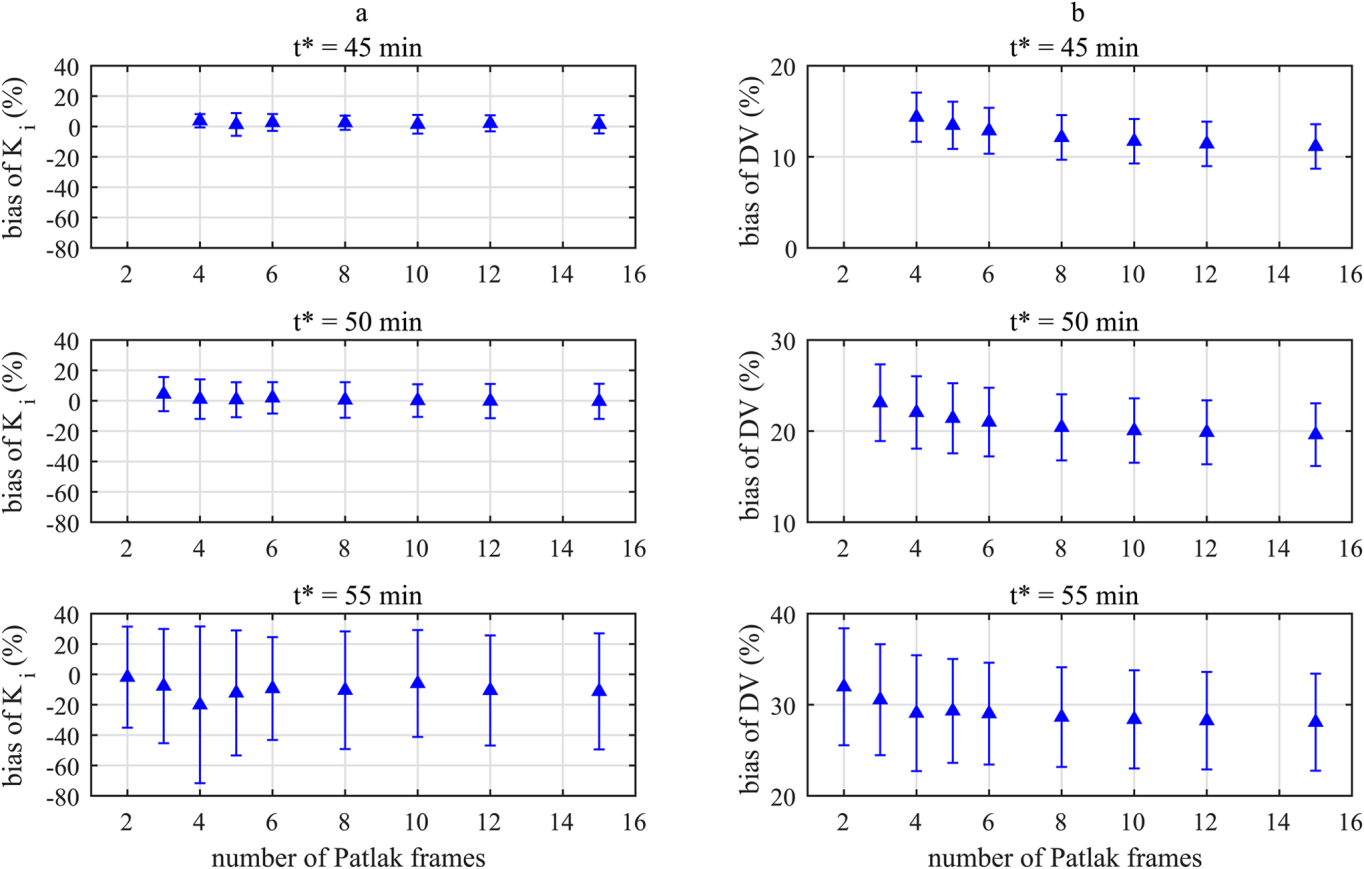
Bias and precision for K_i_ (**a**) and DV (**b**) values for indirect Patlak with sPBIF, both sensitivity modes, t*=45, 50 and 55 min with reference to IDIF based estimation (t*=45 min, 8 Patlak frames)

Independent of the number of Patlak frames, the bias and precision were degrading for K_i_ and DV estimates for increasing t*, reported at Fig. 5. Further an overall tendency was observed for higher number of Patlak frames to result in lower bias and more stable precision for both K_i_ and DV.

The precision of K_i_ was best for 4 frames (3.8 ± 4.4%, t*=45 min), 6 frames (1.9 ± 10.4%, t*=50 min), 2 frames (-1.9 ± 33.3%, t*=55 min), and worst for 5 frames (1.3 ± 7.5%, t*=45 min), 4 frames (1.1 ± 13.0%, t*=50 min), 4 frames (-20.1 ± 51.6%, t*=55 min). Analog the best DV estimates were for 15 frames (11.1 ± 2.4%, t*=45 min), 15 frames (19.6 ± 3.4%, t*=50 min), 15 frames (28.1 ± 5.3%, t*=55 min), and worst for 4 frames (14.4 ± 2.7%, t*=45 min), 3 frames (23.1 ± 4.2%, t*=50 min), 2 frames (32.0 ± 6.4%, t*=55 min).

### Impact of Patlak frames on IDIF based direct Patlak for both sensitivity modes

For the direct Patlak method employing the patient individual IDIF a slightly better bias and precision was obtained with the UHS mode in comparison to HS mode (K_i_: *p* < 0.001 and DV: *p* < 0.001) (Table 3). Similar for the number of Patlak frames only a small impact on K_i_ (*p* = 0.058) and DV (*p* = 0.022) was observed (Table 3).

**Table 3:** Bias and precision for K_i_ and DV values for direct Patlak with patient-individual IDIF, both sensitivity modes, t*=45 min and 5 and 6 Patlak frames with reference to 4 Patlak frames

Number of Patlak frames	Sensitivity mode	bias ± precision (K_i_)	bias ± precision (DV)
5	HS	-1.9 ± 2.7%	2.7 ± 4.1%
	UHS	-0.3 ± 1.6%	-0.1 ± 2.8%
6	HS	-1.1 ± 2.4%	0.8 ± 3.7%
	UHS	-0.1 ± 1.8%	-0.5 ± 2.5%

### Impact of Patlak frames on sPBIF based direct Patlak for both sensitivity modes and abbreviated scan durations

**Fig. 6:**
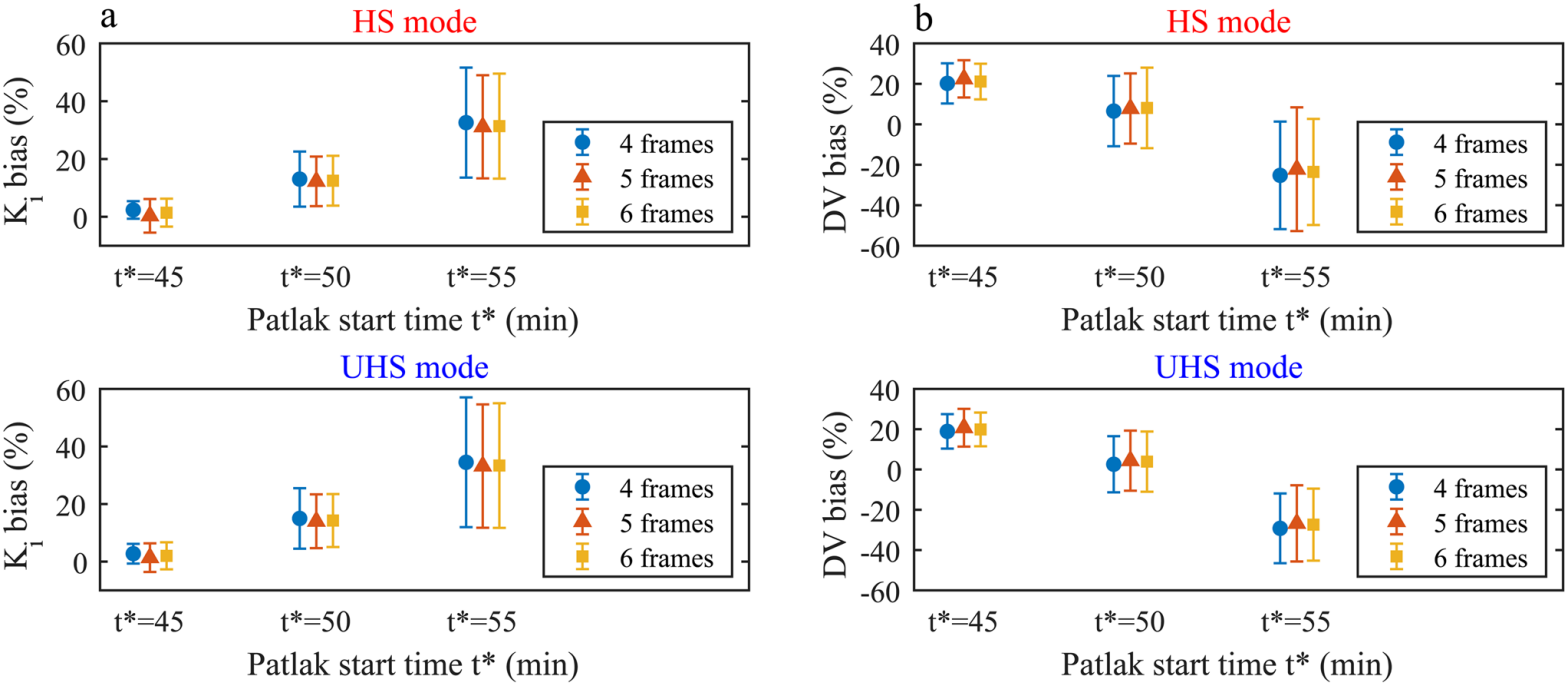
Bias and precision for K_i_ (**a**) and DV (**b**) values for direct Patlak with sPBIF, both sensitivity modes, different number of Patlak frames and t*=45, 50 and 55 min with reference to IDIF based estimation with the same settings

The bias and precision in K_i_ and DV estimates were comparable for both sensitivity modes and for all number of Patlak frames (Supplemental Table 1). A reasonable trade-off between low bias and high precision for both K_i_ (2.7 ± 3.4%) and DV (18.1 ± 7.5%) was obtained for the UHS mode with 4 Patlak frames. Bias and precision are degrading for K_i_ and DV for both sensitivity modes towards higher t*, e.g., for 4 Patlak frames, UHS mode K_i_ from 2.7 ± 3.4%, 15.0 ± 10.5% to 34.5 ± 22.5% and DV from 18.1 ± 7.5%, 2.6 ± 13.9% to -29.2 ± 17.3% for t* of 45, 50 and 55 min, respectively see Fig. 6.

### Direct Patlak reconstructed images comparison using t*=45,50,55 for UHS mode)

**Fig. 7:**
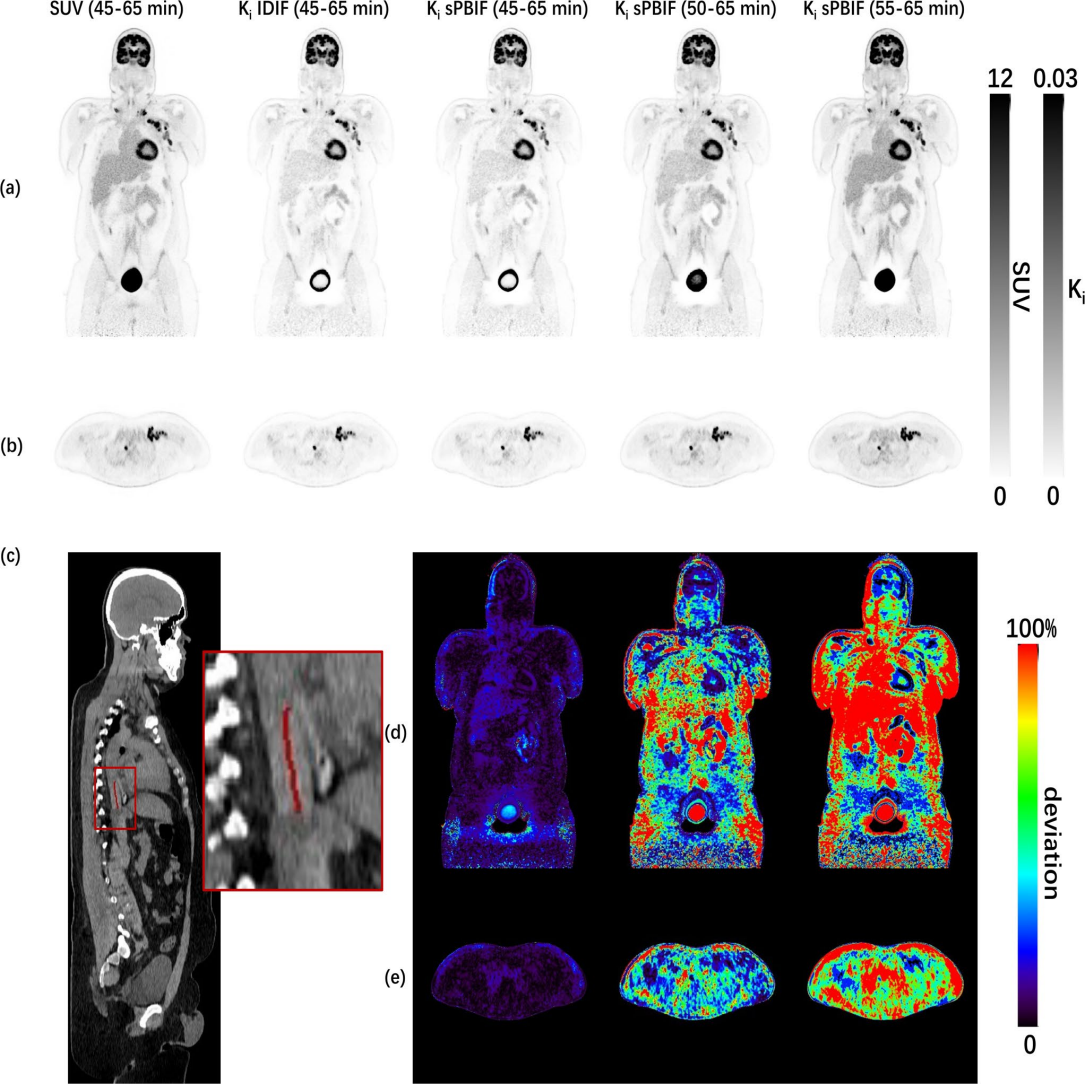
Coronal (**a**) and transversal (**b**) views for SUV and direct Patlak K_i_ images for abbreviated scan times in UHS mode. Sagittal (**c**) view of CT image with 1.68 cm^3^ aortic VOI for IDIF in the thoracic aorta obtained by SnakeVOI. Coronal (**d**) and transversal (**e**) views of absolute relative difference image of K_i_ estimates (left to right: sPBIF (t*=45 min, 45–65 min p.i.), sPBIF (t*=50 min, 50–65 min p.i.) and sPBIF (t*=55 min, 55–65 min p.i.)) with reference to patient-individual IDIF (t*=45 min, 45–65 min p.i.)

The comparison of the SUV and K_i_ images shows a higher contrast for the parametric image (Fig. 7a,b).

This is in accordance with the higher TBR obtained by direct Patlak K_i_ estimate IDIF 17.2 ± 9.6 compared with 6.2 ± 3.1 for the SUV image in UHS mode (Table 4). When shortening the scan time, the TBR of K_i_ is decreased to 6.9 ± 3.5 (55–65 min p.i., UHS mode) from 17.2 ± 9.6 (45–65 min p.i., UHS mode). The TBR was slightly lower for the UHS mode in comparison to HS mode (*p* = 0.01).

**Table 4:** Tumour-to-background ratio (TBR) for SUV and parametric images (direct Patlak IDIF and sPBIF) for both sensitivity modes

	Input function	HS mode	UHS mode
SUV (60–65 min p.i.)	–	6.4 ± 3.1	6.2 ± 3.1
K_i_ (45–65 min p.i. t* = 45 min)	IDIF	18.0 ± 9.0	17.2 ± 9.6
	sPBIF	21.3 ± 11.5	20.2 ± 12.0
K_i_ (50–65 min p.i. t* = 50 min)	sPBIF	11.2 ± 5.6	10.6 ± 5.4
K_i_ (55–65 min p.i. t* = 55 min)	sPBIF	7.2 ± 3.5	6.9 ± 3.5

The similarity of the IDIF and sPBIF (45–65 min p.i.) based Patlak K_i_ estimates is not only visible in Fig. 7, but also, e.g., for the UHS mode a low absolute RC of 0.8 ± 0.3% and high PSNR of 75.7 ± 4.1 (Table 5).

**Table 5:** Non-absolute and absolute relative change (RC), structural similarity index measure (SSIM) and peak signal-to-noise ratio (PSNR) of whole body [^18^F]-FDG K_i_ and DV images using sPBIF

		Sensitivity mode	RC (%)	Absolute RC (%)	SSIM	PSNR (dB)
K_i_	45–65 min p.i.	HS	0.2 ± 0.2	0.8 ± 0.4	0.999 ± 0.001	75.5 ± 3.9
		UHS	0.3 ± 0.3	0.8 ± 0.3	0.999 ± 0.001	75.7 ± 4.1
	50–65 min p.i.	HS	6.8 ± 2.4	7.3 ± 2.4	0.999 ± 0.001	64.5 ± 3.2
		UHS	6.9 ± 2.3	7.5 ± 2.5	0.999 ± 0.001	64.5 ± 3.3
	55–65 min p.i.	HS	18.6 ± 5.7	19.2 ± 5.8	0.997 ± 0.002	59.0 ± 2.7
		UHS	19.1 ± 6.1	19.7 ± 6.2	0.997 ± 0.002	59.2 ± 2.7
DV	45–65 min p.i.	HS	1.5 ± 0.9	1.6 ± 0.8	0.997 ± 0.002	73.1 ± 9.8
		UHS	1.4 ± 0.7	1.5 ± 0.7	0.997 ± 0.002	72.4 ± 9.0
	50–65 min p.i.	HS	0.4 ± 3.6	6.6 ± 2.9	0.953 ± 0.027	61.7 ± 11.4
		UHS	0.1 ± 3.1	6.3 ± 2.4	0.956 ± 0.026	61.2 ± 10.6
	55–65 min p.i.	HS	0.2 ± 7.4	13.7 ± 6.3	0.898 ± 0.036	56.6 ± 10.7
		UHS	-0.1 ± 6.8	13.6 ± 5.7	0.903 ± 0.035	56.0 ± 9.7

Based on the results in Table 5, difference for K_i_ and DV estimates increase towards shorter scan durations, e.g., for HS mode absolute RC of 0.8 ± 0.4%, 7.3 ± 2.4% and 19.2 ± 5.8% (K_i_) and 1.6 ± 0.8%, 6.6 ± 2.9% and 13.7 ± 6.3% (DV) for 20, 15 and 10 min scan duration. The same trend applied to PSNR which was reduced for shorter scan durations both for K_i_ and DV, e.g. for HS from 75.5 ± 3.9 dB (K_i_) and 73.1 ± 9.8 dB (DV) to 59.0 ± 2.7 dB (K_i_) and 56.0 ± 9.7 dB (DV) for 20 and 10 min scan duration, respectively. For SSIM this reduction was only observed for the DV estimates. The larger difference for shorter scan durations could also be observed in Fig. 7d. In particular, K_i_ estimates in the bladder and thorax region deviate largely (RC > 10%) from the reference (IDIF) for shorter scan durations. Accuracy for K_i_ and DV estimates based on sPBIF in comparison to IDIF showed no significant difference between both sensitivity modes (K_i_: *p* = 0.44, DV: *p* = 0.44), e.g., for 50–65 min p.i. absolute RC of 7.3 ± 2.4 and 7.5 ± 2.5 (K_i_) and 6.6 ± 2.9 and 6.3 ± 2.4 (DV) for HS and UHS mode, respectively. Similar no difference in SSIM (K_i_: *p* = 0.61, DV: *p* = 0.02) and PSNR (K_i_: *p* = 0.20; DV: *p* = 0.15) for the different sensitivity modes was observed.

## Discussion

In this study, we evaluated the impact of the sensitivity mode (axial acceptance angle) of the Biograph Vision Quadra LAFOV PET/CT scanner on dynamic imaging with [^18^F]-FDG to determine Patlak K_i_ and DV estimates obtained by the indirect or direct Patlak method. Different Patlak start times t*, number and duration of PET data frames were evaluated to optimise the imaging protocol and analysis settings, and to assess the potential for abbreviated scan protocols using a population-based input function.

### Indirect Patlak

For the indirect Patlak approach using the full IDIF (0–65 min), only minor differences of 3.4 ± 7.0% (K_i_) and 1.2 ± 2.6% (DV) were found between the sensitivity modes. These differences cannot be caused by variations in the IDIF, as the AUC showed excellent agreement, presumably because the number of events for the aortic VOI in each frame was already sufficiently high in the HS mode. Therefore, the difference was caused by the determined activity concentration in the delineated lesion VOI, which could be altered either by the slightly degraded spatial resolution of the UHS mode [23] or by its higher event statistics. Although the largest difference between the two modes is known to be in the centre FOV, no correlation was observed between axial lesion position and differences in Patlak estimates.

However, for the indirect Patlak, the UHS mode was less affected by the number of Patlak frames used to determine K_i_ estimates, e.g., a deviation for 8 Patlak frames of 2.3% (UHS) and 4.8% (HS) compared to 4 Patlak frames. The rationale for increasing the number of Patlak frames was to have more samples for a more accurate Patlak fit, which is less susceptible to variation in individual fit points. Of note, the bias was negative and fairly constant up to 20 Patlak frames (Fig. 3a), indicating that with 4 Patlak frames the true K_i_ value might be overestimated. Therefore, 8 Patlak frames were determined as the optimal setting for the indirect Patlak method.

For the abbreviated protocols based on the sPBIF and indirect Patlak, the UHS mode resulted in a lower bias and higher precision for K_i_ compared to the HS mode, e.g., for 45–65 min p.i. 6.4 ± 8.9% (HS) and 3.8 ± 4.4% (UHS).

Comparison of these results with previous studies on the Biograph Vision Quadra using the HS mode confirms this improvement due to the UHS mode, such as a higher precision of 4.4% compared to 13% (45–65 min p.i.) reported by Sari et al. [20] and a lower bias of 3.8% compared to 5.18% (40–60 min p.i.) reported by Sluis et al. [21]. The more accurate determination of K_i_ with the UHS mode demonstrated in our work allows the scan protocol to be further shortened while maintaining a higher precision than in the HS mode, e.g. 50–65 min p.i. 4.4 ± 11.2% (UHS) instead of 7.3 ± 20.0% (HS).

In contrast, the impact of sensitivity mode on DV was negligible, e.g., 45–65 min p.i. 14.2 ± 2.7% (HS) and 14.4 ± 2.7% (UHS). The best estimates of DV with sPBIF indirect Patlak were obtained with 8 or more Patlak frames. However, K_i_ estimates only showed a minor dependency on the number of Patlak frames between 4 and 15 for 45–65 min and 50–65 min. For the shortest scan duration examined, 55–65 min, a stronger dependence on the number of Patlak frames and poor K_i_ estimates were observed, e.g., -1.9 ± 33.3% (2 frames) and − 20.1 ± 51.6% (4 frames), rendering the use of such short protocols questionable.

### Direct Patlak

The indirect Patlak method with ROI kinetic modelling used in this work is a conventional and easy to implement approach to derive tracer kinetics. However, parametric images, which estimate kinetic parameters for each voxel, are more suitable for studying heterogeneous tracer uptake [27]. Although parametric images can be obtained using the indirect Patlak method, in this work we focused on evaluating parametric images obtained using the direct Patlak method. This was done because it has been shown that direct incorporation of the Patlak model into the image reconstruction process allows accurate noise modelling and results in better bias-variance characteristics than those obtained by indirect methods [32, 33]. In particular for the Biograph Vision Quadra, Sari et al. [34] showed that K_i_ images generated using the direct Patlak method had a twofold higher contrast-to-noise ratio in tumour lesions and yielded 27% higher SNR on average compared to images generated using the indirect Patlak method.

As previously reported for the indirect Patlak with UHS mode, the number of Patlak frames had a smaller impact on K_i_ estimates as for HS mode. This effect was mitigated for the direct Patlak, e.g., for 6 Patlak frames bias and precision of K_i_ estimate was − 1.1 ± 2.4% (HS) and − 0.1 ± 1.8% (UHS) compared to 4 Patlak frames. Similarly, for the sPBIF direct Patlak method, only a negligible difference in K_i_ and DV estimates was observed between the number of Patlak frames. In addition to the reported performance of the UHS and HS mode, another factor is the longer image reconstruction time for the UHS mode. The larger amount of line of responses and event data increases this time, e.g. for the dedicated workstation used in our study from 50 ± 3 min (HS) to 79 ± 3 min (UHS) (average time for a single patient direct Patlak reconstruction including 4 frames for sPBIF generation), which should be considered especially in busy clinical scanning schedules.

The comparison between indirect and direct Patlak showed comparable results for the variation of K_i_ and DV estimates for scan protocols of 20 min duration: e.g., UHS mode 3.8 ± 4.4% and 2.7 ± 3.4% (K_i_) and 14.4 ± 2.7% and 18.2 ± 7.5% (DV) for indirect and direct Patlak, respectively. For shorter scan protocols of 15 min the indirect Patlak showed a lower bias for K_i_ (4.4 ± 11.2% versus 15.0 ± 10.5%) and better precision for DV (23.1 ± 4.2% versus 2.6 ± 13.9%) compared to the direct Patlak. Of note, these measures are not suitable for assessing which method provides the more accurate K_i_ and DV estimates. Instead, due to the lack of a ground truth for K_i_ and DV, they indicate how robust the estimates were within each method for the abbreviated scan durations.

A major advantage of the direct Patlak method is that it yields parametric images with superior contrast in comparison to standard SUV images, e.g., a TBR of 17.2 ± 9.6 (direct Patlak) in comparison to 6.2 ± 3.1 (SUV).

The TBR obtained with direct Patlak and sPBIF was not affected by the sensitivity mode, however degraded towards shorter scan durations 10.6 ± 5.4 (15 min) and with 6.9 ± 3.5 for the 10 min scans did not show any improvement over the TBR obtained by the SUV image.

The K_i_ and DV estimate images showed excellent agreement and image quality (RC: 0.3 ± 0.3% (K_i_), 1.4 ± 0.7% (DV), SSIM: 0.999 ± 0.001 (K_i_), 0.997 ± 0.002 (DV), PSNR:75.7 ± 4.1 dB (K_i_), 72.4 ± 9.0 dB (DV)) when comparing the sPBIF based method to the IDIF method for a scan duration of 45–65 min.

In comparison to the K_i_ related results reported by Sari et al. [20] for a 30 min scan duration with t*=35 min, RC was comparable (0.31 ± 0.25% vs. our work 0.3 ± 0.3%) and PSNR indicated an increased image quality (64.03 ± 3.59dB vs. our work 75.7 ± 4.1dB).

It should be noted, that the quantitative indexes SSIM and PSNR are mainly used for image quality assessment for image processing and computer vision, however these metrics are not necessarily well aligned with human perception [35] therefore a conclusion about clinical impact based only on these metrics is questionable.

However, they are a valid measure to detect differences between images in terms of contrast, structure and noise. Therefore, no difference in these measures for the parametric images obtained either in HS or UHS mode is indicated by the small difference in SSIM and PSNR (SSIM difference < 0.1 and PSNR difference < 1dB).

The higher event statistics obtained with the UHS mode are associated with a lower image noise. In addition, the small degradation of the spatial resolution by the increased parallax error due to more oblique line of response facilitates blurring of smaller structures. No differences in TBR were observed in our work, as the impact on the mean values in K_i_ and DV of the large homogeneous region in the liver was negligible.

The image metrics RC, SSIM and PSNR were determined based on the comparison of sPBIF and IDIF based direct Patlak for the respective mode. Therefore, image noise related to event statistics is present to the same amount in the reference image and no noise induced differences in these metrics were observed.

The estimate images deviated more from the reference measurement with full IDIF towards shorter scan durations. Hence, good agreement and image quality (RC: 6.9 ± 2.3% (K_i_), 0.1 ± 3.1% (DV), PSNR: 64.5 ± 3.3 dB (Ki), 61.2 ± 10.6 dB (DV)) could be obtained for an abbreviated scan protocol from 50 to 65 min p.i.

### Limitations and outlook

Of note, in Fig. 7a, a halo shaped artefact around the bladder was clearly visible for the K_i_ estimate (45–65 min, t*=45 min) and less pronounced for the K_i_ estimate (50–65 min, t*=50 min). This can be explained by the non-linear pattern of the Patlak plot for the bladder and considerable impact of t* on the determination of bladder parametric estimates which results in the Patlak model not being feasible for the bladder [36]. In general, the feasibility of compartmental models for the bladder is questionable, as shown by the inapplicability of one, and two tissue irreversible and reversible compartmental models by Wu et al. [37].

The TBR in Table 4 was 18.0 ± 9.0 and 21.3 ± 11.5 for the IDIF- and sPBIF-based methods, respectively. This difference was caused by an increase in lesion K_i_ (+ 2.7 ± 3.3% for sPBIF) together with a decrease in K_i_ determined for liver VOI (-6.9 ± 2.1 for sPBIF). Furthermore, with the Patlak approach in this work, we used a simplified compartmental model for the liver with a single arterial blood input function. Instead, to account for the dual blood supply from both hepatic artery and portal vein, dual blood supply input functions with reversible compartmental models should be considered for a better approximation of liver kinetics [38, 39]. Similarly, a more comprehensive model for the lung should be considered accounting for the effects of regional lung aeration, blood volume, and water on [^18^F]-FDG uptake [40].

Although in this study scan protocols were already abbreviated to 20 min or less, respiratory and whole-body motion can degrade image quality and quantification accuracy. The impact of motion for total-body PET parametric imaging [41] and advanced methods for patient motion correction of dynamic protocols [42, 43] have been reported. As shown by Sundar et al. [43] by using a diffeomorphic approach for motion correction the volume mismatch across dynamic frames introduced through motion artefacts could be reduced by about 50%. Further by using an advanced parametrization of motion fields in between frames as diffeomorphism, Sun et al. [42] could not only obtain an average improvement in tumor SUV_mean_ of 5.35 ± 4.92% but also for parametric imaging studies a reduction of 11.8% of inter-subject variability in K_i_ quantification of organs.

We plan to incorporate and access the impact of motion correction for whole-body motion [43] as well as respiratory motion [44] on the 20 min short scan protocols in our future work.

In addition, the number of patients (*n* = 6) and lesions (*n* = 26) in this study is limited due to the long PET imaging protocol of 65 min. This complicates the integration of this study protocol into the tight clinical scan schedule and is associated with considerable discomfort for the patients. Additionally, the new Biograph Vision Quadra TB-PET scanner is not yet widely available, which complicates multi-center studies to increase the number of patient scans. Similar to our case, these limitations have also led other groups working on parametric imaging protocols for TB-PET scanners to evaluate their research on a small number of patients and lesions, such as reported by Sari et al. [20], Sluis et al. [21] and Wu et al. [9] with a number of patients (*n* = 8, 12 and 7) and a number of lesions (*n* = 34, 20, 26), respectively. Including more datasets for testing is one of our future work and based on our work, we aim to overcome the limitation in statistics by introducing abbreviated scan protocols of less than 20 min into the clinical routine, which we have already started in our institution for a dedicated patient cohort.

## Conclusions

In this study, using the Biograph Vision Quadra LAFOV PET/CT scanner, we demonstrated that the impact of the sensitivity mode (i.e. acceptance angle of LOR) on parametric imaging for a dynamic [^18^F]-FDG protocol is negligible if patient individual IDIFs were used. However, for abbreviated scan protocols using a population-based input function and the indirect Patlak method, the precision of K_i_ estimates was improved by the ultra-high sensitivity (UHS) mode, enabling scan durations of 15 min (50–65 min p.i.) with a K_i_ estimate bias and precision of 4.4 ± 11.2% compared to the total acquisition of 65 min. The potential to shorten scan duration to 15 min was also demonstrated for the direct Patlak method with K_i_ and DV estimates bias and precision of 15.0 ± 10.5% and 2.6 ± 13.9%, respectively. Overall, our findings indicate a minor impact of the number of Patlak frames on all analyses, suggesting that 8 (indirect Patlak) and 4 (direct Patlak) frames are optimal for abbreviated scans as short as 50–65 min p.i. This study not only advances the understanding of the Biograph Vision Quadra scanner’s capabilities in terms of parametric imaging but also enables shorter, hence more patient friendly scan protocols suitable to be integrated into clinical routine.

### Electronic supplementary material

Below is the link to the electronic supplementary material.

Supplementary Material 1

Supplementary Material 2

Supplementary Material 3

Supplementary Material 4
