# Supplementary material for: Optimization and impact of sensitivity mode on abbreviated scan protocols with population-based input function for parametric imaging of [18F]-FDG for a long axial FOV PET scanner
Source: Eur J Nucl Med Mol Imaging. 2024 May 20;51(11):3346–59. doi: 10.1007/s00259-024-06745-3 (PMC11368996; doi:10.1007/s00259-024-06745-3)
Supplement: Supplementary file 1 — Supplementary Material 1 [file 259_2024_6745_MOESM1_ESM.docx]

Supplementary Materials


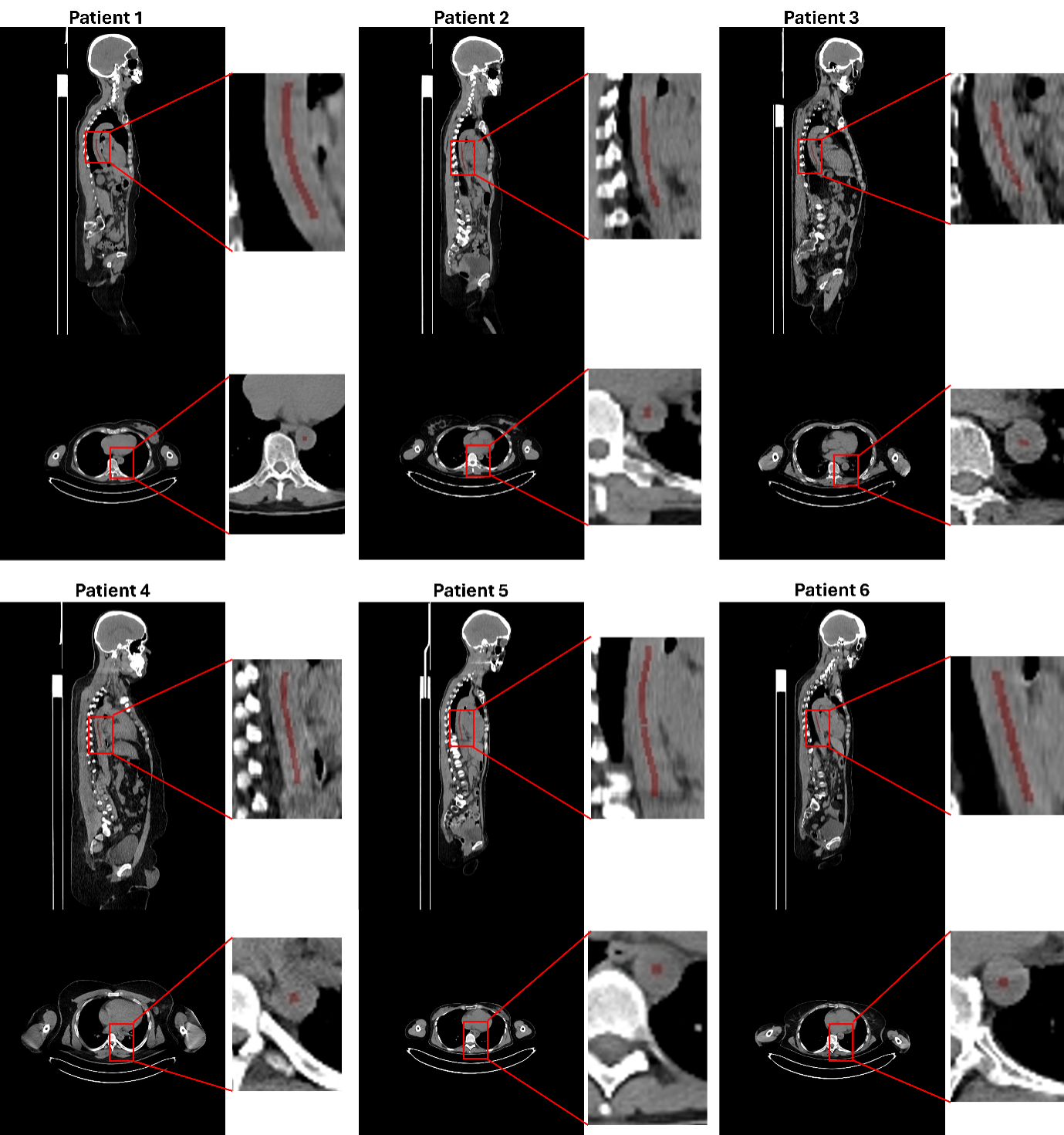


Supplemental Figure 1: Sagittal and transversal view of CT images for all six patients with aortic VOI for IDIF in the thoracic aorta obtained by snake VOI.


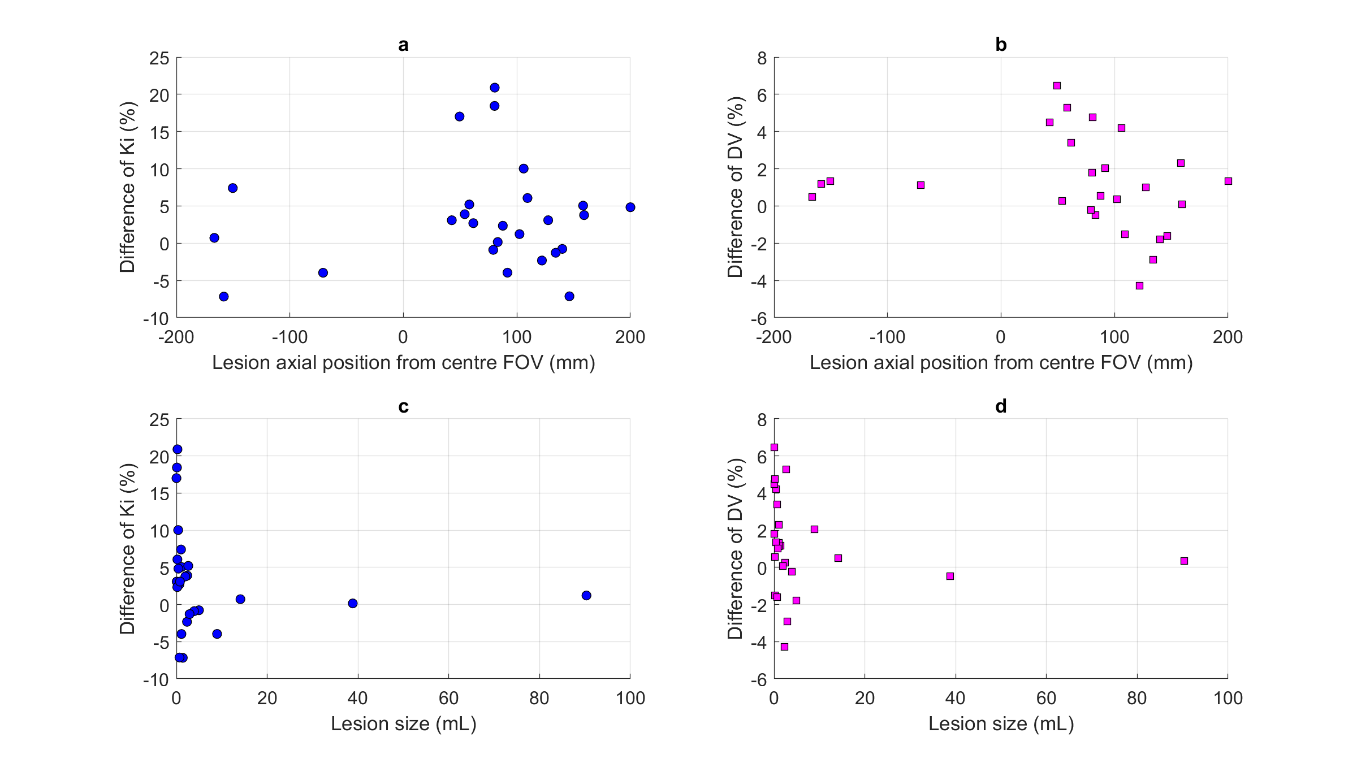


Supplemental Figure 2: Difference of K_i_ and DV values obtained by HS and UHS mode over the axial position (a,b) and lesion size (c,d).


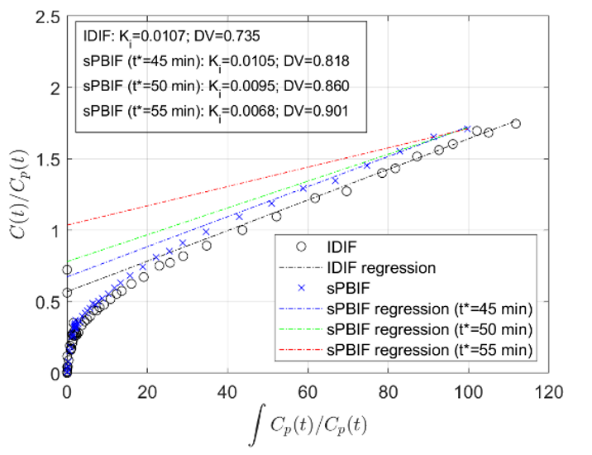


Supplemental Figure 3: Patlak fits for IDIF (t*=45min) and sPBIF (t*=45min, 50 min, 55 min) in UHS mode derived from lesion c(t) and blood pool c_p_ (t) activity concentration over time.

Supplemental Table 1 Bias and precision for K_i_ and DV values for direct Patlak with sPBIF, both sensitivity modes, t*=45, 50 and 55 min, 4-6 Patlak frames and reference to IDIF based method with the same settings.

| t* | Number of Patlak frames | Sensitivity mode | bias ± precision (K_i_) | bias ± precision (DV) |
| --- | --- | --- | --- | --- |
| 45 min | 4 | HS | 2.4 ± 3.0% | 20.2 ± 9.9% |
|  |  | UHS | 2.7 ± 3.4% | 18.1 ± 7.5% |
|  | 5 | HS | 2.2 ± 3.6% | 19.4 ± 9.0% |
|  |  | UHS | 1.6 ± 3.9% | 20.2 ± 8.8% |
|  | 6 | HS | 2.5 ± 3.2% | 20.4 ± 10.7% |
|  |  | UHS | 2.1 ± 3.6% | 19.9 ± 8.4% |
| 50 min | 4 | HS | 13.0 ± 9.5% | 6.5 ± 17.4% |
|  |  | UHS | 15.0 ± 10.5% | 2.6 ± 13.9% |
|  | 5 | HS | 12.3 ± 8.6% | 7.8 ± 17.3% |
|  |  | UHS | 14.0 ± 9.3% | 4.3 ± 14.9% |
|  | 6 | HS | 12.5 ± 8.6% | 8.1 ± 19.9% |
|  |  | UHS | 14.3 ± 9.2% | 3.9 ± 15.0% |
| 55 min | 4 | HS | 32.6 ± 19.0% | 25.2 ± 26.6% |
|  |  | UHS | 34.5 ± 22.5% | 29.2 ± 17.3% |
|  | 5 | HS | 31.1 ± 17.8% | 22.2 ± 30.6% |
|  |  | UHS | 33.2 ± 21.4% | 26.8 ± 18.9% |
|  | 6 | HS | 31.4 ± 18.2% | 23.5 ± 26.2% |
|  |  | UHS | 33.4 ± 21.7% | 27.4 ± 17.9% |
